# Effect of hearing aids use on speech stimulus decoding through speech-evoked ABR^☆^

**DOI:** 10.1016/j.bjorl.2016.11.002

**Published:** 2016-12-08

**Authors:** Renata Aparecida Leite, Fernanda Cristina Leite Magliaro, Jeziela Cristina Raimundo, Mara Gândara, Sergio Garbi, Ricardo Ferreira Bento, Carla Gentile Matas

**Affiliations:** a Universidade de São Paulo (USP), Curso de Fonoaudiologia, São Paulo, SP, Brazil; b Universidade de São Paulo (USP), Fundação de Otorrinolaringologia do Hospital das Clínicas, Ambulatório de Saúde Auditiva Reouvir, São Paulo, SP, Brazil

**Keywords:** Auditory evoked potentials, Hearing loss, Child, Hearing aids, Hearing, Potenciais evocados auditivos, Perda auditiva, Criança, Auxiliares de audição, Audição

## Abstract

**Introduction:**

The electrophysiological responses obtained with the complex auditory brainstem response (cABR) provide objective measures of subcortical processing of speech and other complex stimuli. The cABR has also been used to verify the plasticity in the auditory pathway in the subcortical regions.

**Objective:**

To compare the results of cABR obtained in children using hearing aids before and after 9 months of adaptation, as well as to compare the results of these children with those obtained in children with normal hearing.

**Methods:**

Fourteen children with normal hearing (Control Group – CG) and 18 children with mild to moderate bilateral sensorineural hearing loss (Study Group – SG), aged 7–12 years, were evaluated. The children were submitted to pure tone and vocal audiometry, acoustic immittance measurements and ABR with speech stimulus, being submitted to the evaluations at three different moments: initial evaluation (M0), 3 months after the initial evaluation (M3) and 9 months after the evaluation (M9); at M0, the children assessed in the study group did not use hearing aids yet.

**Results:**

When comparing the CG and the SG, it was observed that the SG had a lower median for the V–A amplitude at M0 and M3, lower median for the latency of the component V at M9 and a higher median for the latency of component O at M3 and M9. A reduction in the latency of component A at M9 was observed in the SG.

**Conclusion:**

Children with mild to moderate hearing loss showed speech stimulus processing deficits and the main impairment is related to the decoding of the transient portion of this stimulus spectrum. It was demonstrated that the use of hearing aids promoted neuronal plasticity of the Central Auditory Nervous System after an extended time of sensory stimulation.

## Resumo

**Introdução:**

As respostas eletrofisiológicas obtidas com o Potencial Evocado Auditivo de Tronco Encefálico complexo (Peate-c) fornecem medidas objetivas do processamento subcortical dos estímulos de fala e outros estímulos complexos. O Peate-c também vem sendo utilizado para verificar a plasticidade na via auditiva nas regiões subcorticais.

**Objetivo:**

Comparar os resultados do Peate-c obtidos em crianças usuárias de Aparelho de Amplificação Sonora Individual (AASI), antes e após 9 meses da adaptação, bem como comparar os resultados destas crianças com os obtidos em crianças com audição normal.

**Método:**

Foram avaliadas 14 crianças com audição normal (Grupo Controle – GC) e 18 crianças com perda auditiva neurossensorial de grau leve a moderado bilateral (Grupo Estudo – GE), na faixa etária de 7 a 12 anos. As crianças foram submetidas às Audiometrias Tonal e Vocal, Medidas de Imitância Acústica e Peate com estímulo de fala, sendo submetidas às avalições em três momentos diferentes: avaliação inicial (M0), 3 meses após avaliação inicial (M3) e 9 meses após a avaliação inicial (M9), sendo que no M0 as crianças do grupo estudo ainda não faziam uso do AASI.

**Resultados:**

Na comparação entre GC e GE, observou-se que o GE apresentou menor mediana para a amplitude V–A no M0 e no M3, menor mediana para a latência do componente V no M9 e, maior mediana para a latência do componente O no M3 e no M9. Observou-se no GE, redução na latência do componente A no M9.

**Conclusão:**

Crianças com perda auditiva de grau leve a moderado apresentaram déficit no processamento do estímulo de fala sendo que o principal prejuízo está relacionado à decodificação da porção transitória do espectro deste estímulo. Evidenciou-se que o uso do AASI promoveu a plasticidade neuronal do Sistema Nervoso Auditivo Central após um tempo prolongado de estimulação sensorial.

## Introduction

The auditory brainstem response (ABR) is widely used in clinical practice to assess the auditory pathway integrity in the brainstem, as well as the electrophysiological threshold in children and infants. Although of great importance in clinical practice, the ABR with click and tone burst stimuli provides a few information on auditory processing for environmental sounds.^1^

The terminology used in the literature to refer to the electrophysiological responses, captured in the brainstem and elicited by complex sound stimuli, is varied, and thus, there is complex-ABR (cABR), speech-evoked ABR and music-evoked ABR.

The electrophysiological responses obtained with the cABR provide objective measurements of subcortical processing of speech and other complex stimuli.^2^, ^3^ The generation of these responses involves a neural circuit that interacts with cognitive processes and is influenced by top-down processing.^4^

Therefore, cABR corresponds to the neural decoding (or coding) of complex sound stimuli, such as music, speech, environmental sounds, among others. One of the characteristics of the cABR is that the captured electrophysiological responses are similar to the spectrum of the complex acoustic stimulus used, thus reproducing its spectral and temporal characteristics.^4^

Several stimuli can be used to investigate how temporal and spectral characteristics are preserved in ABR; one of the most often studied is the speech stimulus, especially the syllable-evoked (vowel–consonant) responses.^3^

Considering that the acoustic characteristics of the speech stimulus are directly involved in the responses that will arise in the ABR (or speech-evoked ABR), some studies have used syllables in the consonant–vowel format (ex: /da/) with duration of 40 ms, and identified the emergence of seven components (V, A, C, D, E, F and O), which would represent the transient (brief) and sustained characteristics of the acoustic stimulus.^3^, ^5^, ^6^, ^7^ The components V, A and C correspond to the transient characteristics of the stimulus, whereas D, E and F correspond to the sustained part of the stimulus or FFR (frequency following response).^3^

For some authors, speech is represented in the brainstem as follows: components V, A, C, and O are generated by neural mechanisms that reflect the transient characteristics associated with aspects of the speech filter (for instance: point and mode of articulation, and modification in articulators – phonetic information), while components D, E and F are generated by neural mechanisms that encode the source of the stimulus production, such as the fundamental frequency (F0)^8^, ^9^ and the harmonics contained between the consonant–vowel transition.^3^

The components V, A, C represent the beginning of voicing and component O, its end. The V–A complex arises from the consonant/d/onset response,^3^ whereas response O represents the offset (end of stimulus).^9^

According to the literature, cABR is used in research with several populations: Musicians,^10^, ^11^ adults with normal hearing,^5^, ^12^ children and adults with central auditory processing disorder,^6^, ^13^ children with phonological disorders,^14^ stuttering,^15^ among others, as it provides objective data on the auditory processing of complex stimuli. The cABR has also been used to verify the plasticity of the auditory pathway in the subcortical regions.^10^

Speech processing begin in the peripheral auditory pathway and occurs in several structures of the central auditory pathway, finishing in the auditory cortex.^16^ During this trajectory, the inner hair cells have the role of transduction of the mechanical stimulus (sound stimulus) into an electric one, so that it is propagated by the auditory nerve. Thus, the alterations in the peripheral auditory system interfere with the quality of the auditory signal that will be sent to the central auditory nervous system (CANS), impairing auditory discrimination.^17^

The use of cABR in individuals using hearing aids has been suggested in the literature, as this procedure reflects both the sensory and cognitive processes, being able to help in the assessment of the central auditory function, hearing aids adaptation, in addition to monitoring the maturation/plasticity of the auditory pathway after the hearing aids adaptation.^4^, ^9^

Considering that the deprivation of sensory stimuli, such as in hearing impairment, affects the normal development and the connectivity required to form a functional auditory system,^18^ it becomes important to evaluate the central auditory pathway of children with hearing loss.

Therefore, the objective of the present study was to compare the results of ABR with speech stimulus obtained in children users of hearing aids before and after 9 months of its adaptation. We also compared the results of these children with those obtained from children with normal hearing.

## Methods

This is a prospective and longitudinal study, approved by the Research Ethics Committee (no 266512/2013). The study was conducted after the parents or guardians were informed about the research and after the signing of the Free and Informed Consent form. It was also necessary for the child to accept participation in the study, with all the procedures being explained to the child through the Term of Assent.

Thirty-two children participated in the study, aged 7–12 years (mean age: 9 years and 8 months (Control Group) and 9 years and 2 months (Study Group), of both genders, without neurological damage or any impairment that could interfere with the cABR measurements, of which 14 had normal hearing (auditory thresholds within normal limits: ≤15 dBHL at the frequencies of 250, 500, 1000, 2000, 4000, 8000 Hz) (control group – CG) and 18 with bilateral mild to moderate sensorineural hearing loss,^19^ without any previous experience with any type of sound amplification device (study group – SG).

The children were submitted to pure tone and vocal audiometry using the Grason Stadler audiometer, model GSI 61 and supra-aural headphones, model TDH 50; Measures of Acoustic Immittance using the Madsen Zodiac 901 Immittanciometer; cABR with a two-channel device, universal Smart Box Jr™ Smart EP, of the Intelligent Hearing System, calibrated at the hearing level (dBnHL), using in-ear ER3A headphones. The cABR was performed only in the right ear.

To obtain the cABR, the child remained seated in a comfortable position in an acoustically and electrically treated room. The skin was cleaned with abrasive paste and the electrodes were fixed to the skin using electrolytic paste and surgical tape (micropore). The active (Fz) and the ground (Fpz) electrodes were positioned on the forehead, and the reference electrode was positioned on the right mastoid (M2). The positioning of the electrodes was in accordance with the International Electrode System IES 10–20 norms.^20^ The impedance values of the electrodes were verified and were below 5 kΩ. In order to obtain cABR, the synthetic stimulus speech/da/was presented, with duration of 40 ms at 80 dBnNA at a presentation rate of 11.1 stimuli per second, with a total of 3000 stimuli (three scans of 1000 stimuli), with alternating polarity, band-pass filter 100–3000 Hz and with a recording window of 60 ms. The three scans of 1000 stimuli were summed and, in the resulting tracing, the components V, A, C, D, E, F and O were identified and analyzed.

The children were evaluated at three different moments: CG: initial evaluation (M0) and three (M3) and nine (M9) months after the initial evaluation; SG: before the hearing aids adaptation (M0) and three (M3) and nine (M9) months after hearing aids using It should be noted that two children from the SG and one from the CG did not attend all three evaluations.

All assessments were carried out by the same evaluator at the three different times and the analysis of the ABR components was performed by the evaluator and by two referees, considering the consensus analysis. In cases where there was no consensus among the three referees regarding the assessments, the tracings were disregarded and excluded from the sample.

In addition to the tabulation of the latency and amplitude values of the ABR components at each moment of evaluation, the results for each individual were classified as: present (presence of all components) and partial (absence of one or more components). There were no individuals with absence of all components in this sample (Table 1).

**Table 1:** Comparison between the distribution of the occurrence of present and partial responses at each moment for the control and study groups.

Moment	Responses	Control group		Study group		Total
		*N*	%	*N*	%	
MO	Present	9	100	15	93.8	24
	Partial	0	0	1	6.3	1
	Total	9	100	16	100	25
M3	Present	10	100	11	78.6	21
	Partial	0	0	3	21.4	3
	Total	10	100	14	100	24
M9	Present	12	100	12	80	24
	Partial	0	0	3	20	3
	Total	12	100	15	100	27

*N*, sample size.

The statistical analysis showed that data was not normally distributed for all variables and thus, for the descriptive analysis, the median and interquartile range were used. Non-parametric tests were used for inferential analysis. Friedman's ANOVA was used to compare the performance of the individuals at the three moments of assessment (M0, M3 and M9). The Mann–Whitney test compared performance between the groups. The significance level was set at 5% and the significant results were identified with an asterisk (*).

## Results

Table 2 shows that there were no statistically significant differences for the V–A amplitude between all the assessed moments (M0 × M3 × M9), for both the SG and CG.

**Table 2:** Comparison of the V–A amplitude of ABR with speech stimulus, at moments 0, 3, 9 months, in the control (CG) and study (SG) groups.

ABR with speech stimulus		V–A amplitude				
				Percentile interval		
		*N*	Median	1st quartile	2nd quartile	M0 × M3 × M9
CG	M0	9	0.32	0.22	0.57	0.965
	M3	10	0.4	0.25	0.54	
	M9	12	0.34	0.22	0.43	
SG	M0	15	0.2	0.17	0.28	0.895
	M3	13	0.2	0.18	0.3	
	M9	14	0.26	0.21	0.31	

*N*, sample size.

**Figure 1:**
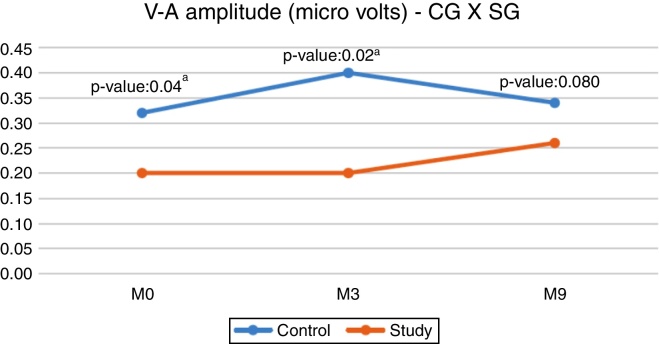
Comparison between the CG and the SG for V–A amplitude. ^a^ Statistical significant difference

When comparing the CG and SG regarding the V–A amplitude, a statistically significant difference was observed in the initial evaluation (M0) and in the second evaluation (M3), with the CG showing a higher median (Fig. 1).

Regarding the CG, no statistically significant differences were observed when comparing the latencies of the ABR components with speech stimulus between the assessed moments (Table 3). However, for SG, there was a statistically significant difference for component A latency between the moments M0 × M9, and at M9, the latency showed a decreased value (Table 4).

**Table 3:** Comparison of ABR latencies with speech stimulus in the control group.

Latency	Moment	*N*	Median	Interval		*p*-Value
				1st quartile	3rd quartile	
V	M0	9	6.88	6.44	7	
	M3	10	6.63	5.97	7.41	0.121
	M9	12	6.88	6.35	7.5	
	M0	9	8.25	7.94	9.25	
A	M3	10	8.13	7.35	9.04	0.432
	M9	12	8.32	7.94	9.81	
	M0	9	16.88	15.94	18.32	
C	M3	10	17.63	16.44	19.13	0.396
	M9	12	17.76	16.63	18.35	
	M0	9	22.63	22.26	24.13	
D	M3	10	22.25	21.97	24.04	0.857
	M9	12	22.51	22.25	23.22	
	M0	9	32.5	31.57	34.26	
E	M3	10	31.07	30.72	32.7	0.156
	M9	12	31.82	31	32.85	
	M0	9	40.13	39.69	44.51	
F	M3	10	39.63	39.35	40.38	0.396
	M9	12	39.57	39.25	41.06	
	M0	9	48.5	48.07	51.57	
O	M3	10	47.75	47.35	48.97	0.115
	M9	12	48.63	47.85	49.35	

*N*, sample size.

**Table 4:** Comparison of ABR latencies with speech stimulus in the study group.

Latency	Moment	*N*	Median	Interval		*p*-Value	Comparisons
				1st quartile	3rd quartile		
V	M0	15	6.38	6.13	7		–
	M3	13	6.5	6	7.5	0.97	
	M9	14	6.13	5.91	6.53		
	M0	15	8.63	7.88	8.88		
A	M3	13	7.88	7.75	9.25	0.018^a^	M0 ≠ M9 (*p* = 0.020^a^)
	M9	14	7.88	7.5	8.19		
	M0	15	17.63	16.25	18.88		
C	M3	13	17.38	16.5	19.44	0.748	–
	M9	14	17.13	16.44	17.69		
	M0	16	22.69	21.56	24.16		
D	M3	14	22.51	21.97	24.03	0.922	–
	M9	14	22.19	21.32	23.38		
	M0	16	31.63	30.53	33.76		
E	M3	13	32.25	30.94	33.38	0.347	–
	M9	15	31.5	30.5	34		
	M0	16	40.82	39.25	43.16		
F	M3	14	40.01	39.25	41.94	0.913	–
	M9	14	39.38	39.19	42.16		
	M0	16	51.69	49.47	52.97		
O	M3	14	51	48.88	52.85	0.584	–
	M9	15	51	49	54.13		

*N*, sample size.

a Statistically significant difference (*p* ˂ 0.05) – Friedman's ANOVA.

**Figure 2:**
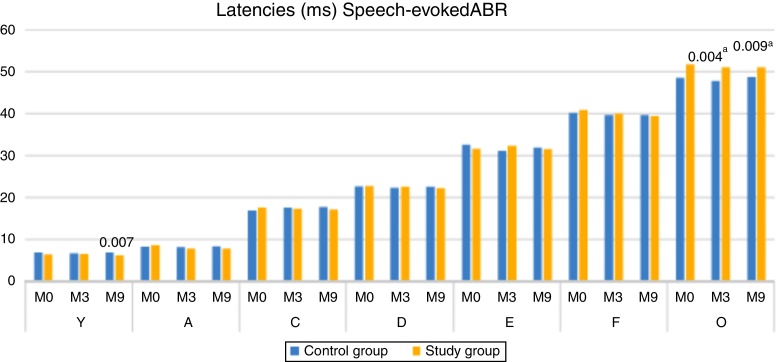
Comparison of Latencies of ABR components with speech stimuli, at moments 0, 3 and 9 months, between the Control and Study Groups–Mann–Whitney. ^a^ Statistical significant difference

When comparing the latencies of ABR components with speech stimulus, between the CG and SG, there was a statistically significant difference for component V at M9 (higher median in CG), and for component O at both M3 and M9, with the SG showing the higher median (Fig. 2).

## Discussion

The cABR has been used in studies with different populations, as it provides objective data regarding the processing of relevant stimuli in the subcortical region of the CANS,^3^ besides being a very important tool in the assessment of the auditory pathway plasticity of these structures.^9^, ^10^

In the present study, when comparing the V–A amplitude between the different assessed moments, we observed that the medians remained similar in both groups, and there was no statistically significant difference between the assessed moments (Table 2). It is worth mentioning that the V–A amplitude refers to the onset burst, that is, the electrophysiological response corresponding to the consonant/d/onset response and, therefore, the groups maintained stability regarding the neuronal response pattern during the 9 months of follow-up.

However, when comparing the CG and the SG, we found differences between the M0 and M3 moments, with the SG showing lower amplitude values (Fig. 1). These results reinforce the idea that an intact and properly functioning auditory pathway (CG) has a greater number of neurons activated by the acoustic stimulus. The result of the present study demonstrated that the deprivation of adequate sensory stimulation, as in hearing impairment, can impair the normal development and the connectivity required to form a functional auditory system. ^18^

Although the SG showed a lower V–A amplitude value at M9, we observed there was no statistically significant difference between the groups, thus demonstrating that there was a change in the central auditory pathway of the SG children after 9 months of hearing aids use, showing that the response to the onset burst (Fig. 1) was close to the responses obtained by children with normal hearing (CG). This modification that occurred after the hearing aids use indicates a possible neuroplasticity of the central auditory system after auditory stimulation due to the use of hearing aids. For Cramer et al. (2011)^21^ neuroplasticity is the ability of the nervous system to reorganize its structures, functions and connections in response to intrinsic and extrinsic stimuli.

Regarding the study of the latencies of the cABR components in each group separately (Table 3, Table 4), there was stability in the latency of the CG components between the three assessed moments (during the 9 months of testing); however, there was a significant decrease in the latency value of component A in the SG between M0 and M9 (Table 4). When assessing the latencies of the other components of the cABR in the SG, a decreased was observed, although without statistical difference.

These findings indicate that the maturational process of the central auditory pathway of children aged 7–12 years during the 9-month period did not have an effect on the cABR results in the group of children with normal hearing (CG). However, the auditory pathway stimulation using hearing aids (SG) for nine months seems to have resulted in a structural/functional change in the neurons responsible for the transmission and decoding of the speech stimulus. This neuroplasticity was demonstrated mainly by the decrease in transmission/decoding time in the structures responsible for wave A generation.

Although they did not use cABR, studies with cortical auditory evoked potential in children with sensorineural hearing loss showed an improvement in the latency values of component P1^18^, ^22^ as well as significant improvement in the morphology of component N2^23^ after acoustic stimulation through a cochlear implant.

When comparing the groups, it was observed that the children of the SG had a lower latency of component V, at M9, when compared to the children of the control group (Fig. 2). The modification of the V–A amplitude (M9), the decrease in the latency of component A in the SG (M9) and the lower latency of component V in the SG (M9), reinforce the occurrence of neuronal plasticity, verified in the improvement of the electrophysiological response after 9 months of auditory stimulation with the hearing aids. These findings also demonstrate that in addition to the importance of the hearing aids use, the time of stimulation was a decisive factor, as the significant changes occurred at M9.

The use of hearing aids possibly promoted greater auditory stimulation and an increase in responsive neuronal fibers, and consequently, new neuronal connections of the subcortical CANS regions. The electrophysiological responses showed significant changes after the ninth month of hearing aids use, showing that the neuronal plasticity process depended on a longer period of auditory stimulation. The findings of the present study demonstrated a lack of agreement with the studies that showed electrophysiological changes after the first month of use of hearing aids.^18^, ^22^ One hypothesis for this discrepant results may be the fact that the aforementioned studies evaluated cortical auditory evoked potentials, whereas in the present study the cABR responses reflect the subcortical processing of the speech stimulus.^2^, ^3^

The results of the present study also showed that the SG had higher latency values of component O at M0, M3 and M9, being statistically significant at M3 and M9. These results suggest that, even with auditory stimulation through hearing aids the children of the SG demonstrated a delay in the central auditory pathway maturation, responsible for the generation of the O component (offset), that is, the structures that decode the end of the syllable (/da/). According to the literature, children with auditory deprivation (profound hearing loss) for more than 7 years do not reach the expected normality for the P1 component even after years of stimulation (cochlear implant), demonstrating a delay in the central auditory pathway maturation.^18^

It should be noted that the results of the present study showed similar results between the groups for some cABR components (A, C, D, E, and F). One of the hypotheses for these findings may be the degree of hearing loss (mild to moderate) of the individuals assessed in this study and, thus, the sensory deprivation was not complete, which led to a lower degree of speech stimulus decoding impairment. Additionally, hearing loss is known to interfere with electrophysiological responses, generally reducing the amplitude and increasing the latency of the components^24^; however, these patterns of responses were not verified for the aforementioned components, although the study group was evaluated with in-ear phones (without the use of hearing aids).

It was observed that the components with the worst results in the SG (V–A amplitude and O latency) are generated by neural mechanisms that reflect the transient characteristics of the speech stimulus spectrum and are associated with aspects of the speech filter (for instance, point and mode of articulation and modification in articulators – phonetic information).^8^, ^9^ These results suggest that children with mild to moderate sensorineural hearing loss have impairment in auditory sensory regions responsible for the phonetic information decoding.

The results of the present study were similar to those found by Johnsons et al.^25^ who evaluated cABR in children with learning disorders. According to the authors, these children showed latency delay of components A, C (beginning of vocalization) and O (end of vocalization), when compared to children with typical development, suggesting that children with learning disorders have poor temporal resolution.^25^

Temporal resolution is the ability of the auditory system to detect small changes (amplitude and spectral content) in the sound stimulus over time,^26^ and it is an important skill for speech discrimination. The perception of the size of the silence interval between the onset and the vowel of one syllable (Voice Onset Time - VOT) is directly associated with the discrimination between two phonemes (ex: /ba/ and /pa/).^27^ Therefore, changes in the processing of fast transient acoustic signals (onset) lead to a poor ability to distinguish phonemes.^28^

In a study with individuals with mild to moderate sensorineural hearing loss, it was observed that this population had a significantly poor performance in temporal resolution.^29^ Therefore, the results found in the present study may suggest that children with mild to moderate sensorineural hearing loss have temporal resolution impairment, as the alterations are concentrated in the beginning of the onset and the end of the vocalization (offset), which correspond to the transient characteristics of the speech stimulus.

In the present study, cABR was shown to be an important tool for the monitoring of auditory processing and auditory system plasticity after stimulation with hearing aids. Studies using this potential can bring important contributions to clinical practice, especially regarding the use of these biological markers in the evaluation of the effectiveness and benefits of hearing aids during the auditory rehabilitation process.

## Conclusions

Children with mild to moderate hearing loss showed impairment in speech stimulus processing, as measured by cABR, when compared to children with normal hearing. The main impairment is related to the decoding of the transient portion of the speech stimulus spectrum (components V–A and O), which may originate from altered temporal resolution.

Additionally, it was verified that the use of hearing aids promoted neuronal plasticity of the CANS, leading to structural and temporal changes in the responses obtained in individuals with hearing loss, after a prolonged sensory stimulation time.

## Conflicts of interest

The authors declare no conflicts of interest.
